# The Role of Selenium During Gestation in the Development of Fetal Congenital Anomalies: A Systematic Review

**DOI:** 10.3390/nu18030479

**Published:** 2026-02-01

**Authors:** Nikolina Stachika, Ermioni Tsarna, Stavroula-Ioanna Kyriakou, Christina Dalla, Anastasios Potiris, Sofoklis Stavros, Panagiotis Christopoulos

**Affiliations:** 1 Second Department of Obstetrics and Gynecology, “Aretaieion” University Hospital, Medical School, National and Kapodistrian University of Athens, 115 28 Athens, Greece; nnikolinast@gmail.com (N.S.); ermioni.tsarna@gmail.com (E.T.); stavrianna.kyr@gmail.com (S.-I.K.); cdalla@med.uoa.gr (C.D.); 2 Third Department of Obstetrics and Gynecology, University General Hospital “ATTIKON”, Medical School, National and Kapodistrian University of Athens, 124 62 Athens, Greece; apotiris@med.uoa.gr (A.P.); sfstavrou@med.uoa.gr (S.S.)

**Keywords:** selenium, pregnancy, congenital anomalies, neural tube defects, orofacial clefts, congenital heart anomalies, urinary tract anomalies, oxidative stress, thyroid function, folate metabolism

## Abstract

**Background/Objectives**: During intrauterine development, cell proliferation, differentiation, and apoptosis are strictly regulated for organogenesis to be ensured; disruption of these processes, e.g., by oxidative stress, may lead to congenital anomalies. This systematic review aimed to examine the role of selenium (Se), an important antioxidant, during gestation in the development of congenital anomalies. **Methods**: To identify relevant original research studies in English, PubMed, Embase, and Cochrane Library were systematically searched up to December 2025. A qualitative synthesis, quality appraisal, and assessment of predefined sources of bias and heterogeneity were performed. **Results**: 2743 titles and abstracts were screened, 473 full texts assessed, and 31 papers included. Selenium exposure did not affect the risk of all/any congenital anomalies (*n* = 20,815), abdominal (*n* = 89,273) and limb anomalies (*n* = 551,547), chromosomal anomalies (*n* = 1242), or fetal alcohol syndrome (*n* = 41). Higher concentrations of Se were associated with increased risk for urinary tract anomalies (*n* = 2150), but decreased risk for congenital heart defects (*n* = 1807), neural tube defects (max *n* = 12,188), and orofacial clefts (max *n* = 1155). **Conclusions**: Available scientific evidence arises from observational studies and is prone to confounding mainly by gestational age, while only one randomized controlled trial has been identified. Given the major contribution of congenital anomalies to neonatal morbidity, mortality, and long-term impairment of quality of life, well-designed prospective studies are required to establish scientific consensus, define optimal maternal Se levels during pregnancy, and provide evidence-based recommendations for Se supplementation during pregnancy.

## 1. Introduction

Selenium (Se) is a trace element and is essential for human health and development. In the environment, Se can be found in soils and waters, both in elemental form and in inorganic compounds, as selenides, selenates, and selenites [1,2,3]. In biological systems, Se is primarily incorporated into the amino acids selenomethionine (SeMet) and selenocysteine (SeCsy), which are components of several proteins and enzymes [2]. To date, 25 selenoproteins containing SeCsy in their active center have been identified, including glutathione peroxidases (GPx), thioredoxin reductases (TRs), and iodothyronine deiodinases (DIs) [3]. Glutathione peroxidases and TRs play key roles in antioxidant defense, while DIs are essential for thyroid hormone activity; selenoprotein-P is the main plasma selenoprotein involved in Se transport and homeostasis [1].

Dietary intake represents the primary source of Se in humans, with concentrations in food reflecting soil Se content [4]. Animal products, seafood, and some vegetables contain relatively high Se levels, although grains and cereals contribute most to the overall intake in many populations [4]. Selenium has a narrow beneficial concentration window, with blood concentrations between 80 and 140 ng/mL being considered safe [2]. Recommended intake values, required to achieve Se levels within this range, vary by organization and physiological state, including pregnancy and lactation [4,5]. Selenium deficiency among pregnant women is relatively common worldwide, with prevalence varying substantially across regions, countries, and socioeconomic strata, largely reflecting geographic differences in soil Se content and dietary intake patterns. In African and Asian populations, Se deficiency has been reported in 36.3% of pregnant women in Nepal, 5–28% in China, and severe deficiency in up to 99% of pregnant women in Zambia [6,7,8,9]. In Europe, a high prevalence of Se deficiency during pregnancy has been documented in Latvia (30%) [10], and Poland (79%) [11]. In contrast, Se deficiency appears to be relatively uncommon among pregnant women in the United States of America [12].

Adequate Se levels support multiple physiological functions, largely through antioxidant and redox-regulating mechanisms. Selenium deficiency has been associated with cardiovascular, endocrine, immune, neurological, and musculoskeletal disorders [1,2,3,4,5,13]. Conversely, excessive Se exposure has been linked to gastrointestinal, hepatic, neurological, reproductive, developmental, and metabolic toxicity in human and animal studies [14]. During intrauterine development, tightly regulated cell proliferation, differentiation, and apoptosis are essential for normal organogenesis, and their disruption may result in congenital anomalies [15]. Oxidative stress is a recognized contributor to teratogenesis, and pregnancy is characterized by increased oxidative burden, particularly during organogenesis, when the embryo relies predominantly on aerobic metabolism and is more vulnerable to oxidative damage [15,16].

As a key component of antioxidant defense systems, particularly through GPx activity, Se may influence the balance between oxidative and anti-oxidative processes during gestation and thereby affect fetal development. Taking into account the high prevalence of Se deficiency during pregnancy, even a modest effect on the risk of congenital anomalies may have a considerable public health impact. The aim of this systematic review is to examine the published scientific evidence regarding the role of Se during gestation in the development of fetal congenital anomalies.

## 2. Materials and Methods

In the Selenium Exposure during Pregnancy Project (SEduP), we aimed to gather and study all the existing scientific evidence regarding the effects of Se status during pregnancy upon several pregnancy and neonatal outcomes and complications that occur during pregnancy or within the first month of offspring life. We aimed to include all original peer-reviewed research articles and posters presented at conferences that examined the effect of Se exposure during pregnancy on birth and neonatal outcomes. Specifically, eligibility for inclusion was considered to be all randomized control trials, cohort studies, cross-sectional, and case-control studies that examined the effect of Se supplementation on pregnant women, as long as the control group consisted of pregnant women as well. Selenium supplementation was not considered necessary in the case of studies measuring the levels of Se in biological samples of participants (e.g., whole blood, serum, plasma, urine, hair, or nails). Regarding the outcomes, for this systematic review we focused on studies that examined the incidence of fetal congenital anomalies. Articles were excluded if they were not written in English, if dietary Se intake was estimated only via food frequency questionnaires, and if the supplemented group received other vitamins and/or minerals except Se that were not administered to the control group. Case reports, literature reviews, systematic reviews and meta-analyses, editorials, letters to the editor, and articles with no full text available were also excluded.

In order to identify eligible articles, the databases of PubMed, Embase, and Cochrane Library were searched up to December 2025. We developed a search algorithm for PubMed, which included keywords and Medical Subject Headings (MeSH) terms related to pregnancy and Se, as well as a term that excluded animal studies without excluding human studies. No keywords or MeSH terms were included with regard to congenital anomalies or other outcomes, as this search was applied for the SEduP and not only for the present systematic review (Table 1). The search algorithm was translated through Polyglot [17] from Systematic Review Accelerator for Embase (Table S1) and Cochrane Library (Table S2) [18].

All records retrieved through the aforementioned literature search strategy were imported in Rayyan, an online artificial intelligence tool used to simplify and organize the screening of articles in systematic reviews [19]. In this stage, some articles were detected as duplicates and were excluded after manually confirming duplication. The abstracts and titles from all records were independently screened by two reviewers (N.S. and S.-I.K.). Articles that were obviously irrelevant were excluded at this stage. Emerging conflicts were resolved through discussion with a third senior reviewer (E.T.) or were assessed in full text in the case of persisting conflict. The full texts of all potentially relevant records were then sought and uploaded in Rayyan. The full text screening was conducted by the same two reviewers independently (N.S. and S.-I.K.), while conflicts were resolved through discussion and consultation with a third senior reviewer (E.T.). Articles were finally included at this stage if the study protocol met all the inclusion criteria and if the relevant findings were analyzed and presented independently. Lastly, the references of all included studies in this systematic review were manually searched to identify more potentially relevant studies not captured by our search algorithm.

To qualitatively summarize the evidence regarding the effect of Se exposure during pregnancy on congenital anomaly incidence, we extracted and tabulated pre-specified characteristics and findings from all included studies, in a Summary of Findings (SoF) table by one reviewer (N.S.) (Table S3). A second reviewer confirmed the accuracy of data in the SoF table (E.T.). Quantitative synthesis of evidence was regarded as non-feasible, taking into consideration the heterogeneity in study designs and statistical methods used to assess the association between Se status during pregnancy and the risk for fetal congenital anomalies. In particular, we recorded the study’s title with the name of the first author, the year of publication, whether it was a research paper or a conference poster, the study’s geographical region and whether this was urban, suburban, or rural (if specified), the study design, the sample size, type of Se exposure assessment (Se supplementation or measurement of Se levels in biological samples) and timing of assessment, the levels of measured Se in the studied population, details about the examined outcomes (e.g., type of congenital anomaly), the statistical methods applied, and the study’s main findings. Taking this data into consideration, we lastly expressed a simplified conclusion for each study. The studies were further categorized in subgroups, according to the specific type of congenital anomaly studied, namely, all/any congenital anomalies, chromosomal anomalies, neural tube defects, orofacial clefts, congenital heart defects, fetal alcohol syndrome, abdominal congenital anomalies, congenital limb anomalies, and congenital urinary tract anomalies.

Predefined potential sources of bias included the gestational age at sampling and the maternal age, the co-exposure to other elements, smoking, and alcohol habits, and how the aforementioned were accounted for in the analysis (e.g., matching, adjusted statistical models). In addition, to further address potential sources of bias and/or heterogeneity, we recorded the variables, for which results were adjusted, and the statistical method used to this end. The set of potential covariates from studies reporting on similar outcomes were then compared to identify sources of residual confounding in individual studies. Since the reporting on the impact of Se during pregnancy in the development of fetal congenital anomalies was among our inclusion criteria for this systematic review, reporting bias could not be explored; any study that may have examined but did not report on this association was a priori excluded from our systematic review. Timing of sampling (trimester of pregnancy or gestational week) was recorded as a potential source of heterogeneity of individual studies’ results and, additionally, to examine whether the measured Se levels reflected well the critical gestational period for the development of the congenital anomaly studied. Lastly, the levels of Se observed in each study’s population were recorded, as heterogeneity in results from individual studies may arise from a non-linear effect of Se exposure and different Se levels among study populations. The aforementioned sources of bias and heterogeneity were discussed extensively in a group meeting.

A formal quality appraisal of the included studies was also performed (Table S4). To this end, we used Cochrane RoB 2 tool for randomized controlled trials (RCTs), a tool developed by Mamluk et al. for mendelian randomization (MR) studies, and Newcastle-Ottawa scale (NOS) for cohort studies and case-control studies, as appropriate [20,21,22]. For comparability assessment, in studies that measured Se levels in biological samples, we regarded gestational age at sampling as the most important factor, with maternal age, smoking, alcohol consumption, and co-exposure to other elements serving as additional factors. For spatial studies examining environmental Se exposure, maternal age was regarded as the most important factor, as it reflects socioeconomic position that correlates with residential area, and alters the risk for congenital anomalies; additional factors were other indicators of socioeconomic position, smoking, and co-exposure to other elements. In quality appraisal of cohort studies, outcome of interest was regarded as not present at start of the study, if sampling took place periconceptionally or during first trimester of pregnancy. Follow-up was regarded not long enough for outcomes to occur, if congenital anomalies were assessed only based on prenatal ultrasounds. For case-control studies, a star was not assigned if case definition was achieved solely by registry linkage, even though this may underestimate study quality in the case of well-designed, validated registries. Similarly, hospital controls were not assigned a star for representativeness, leading to systematic quality underestimation in the case of studies conducted in areas where the vast majority of births take place in hospital setting. To be noted, NOS scale for case-control studies does not address the potential of reversed causality, which can affect the results of case-control studies measuring Se levels after first trimester. With regard to level of certainty in the body of evidence, we have prespecified that assessments would be conducted through consensus discussion among all authors, separately for each subgroup of studies defined by the specific type of congenital anomaly examined. To this end, we applied the Grading of Recommendations Assessment, Development, and Evaluation (GRADE) approach for exposures [23]. Shortly, non-randomized studies, such as cohort and case-control studies, start as high-certainty evidence. Subsequently, certainty of the evidence is down rated based on five domains, namely, risk of bias (up to two levels), inconsistency, indirectness, imprecision, and publication bias. To be noted, inconsistency and publication bias cannot be estimated in the case of outcomes for which a single study is included in this systematic review, potentially leading to over-estimation in the certainty of evidence. The reporting of this systematic review adheres to the Preferred Reporting Items for Systematic Reviews and Meta-Analyses (PRISMA) guidelines (Tables S5 and S6) [24].

## 3. Results

**Figure 1:**
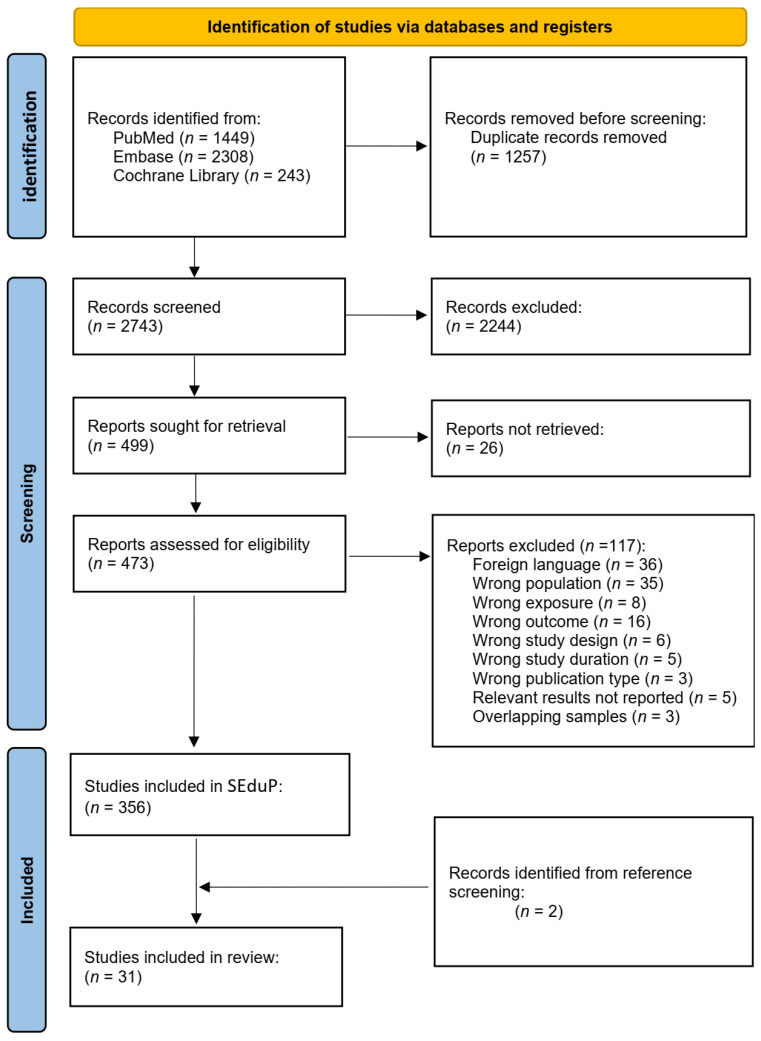
Flow diagram of studies included in the systematic review. SEduP: Selenium Exposure during Pregnancy Project.

By applying our research algorithm, 4000 articles were identified and imported in Rayyan. More specifically, 1449 articles were identified in PubMed, 2308 in Embase, and 243 in Cochrane Library. In this stage, 1257 records were identified as duplicates and were deleted before the screening process. Finally, 2743 records were included in the title and abstract screening process. Among these, 2244 were excluded as obviously irrelevant, while 499 were regarded as eligible for inclusion and were, thus, sought for retrieval. The full text of 26 articles could not be retrieved due to lack of access or because the journal’s archival coverage did not include older issues. Of the remaining 473 articles, 356 are currently included in SEduP based on the full text screening, while 117 were excluded. Specifically, 36 were excluded because they were not written in English, 35 did not include pregnant women, 16 did not examine relevant outcomes, 8 did not examine Se exposure during pregnancy, 6 did not measure the effect of Se independently, 5 surpassed the accepted time frame (one month after birth), 3 were not among the included publication types, 5 studies examined relevant outcome but did not report the relevant findings, and, lastly, 3 studies were excluded because the same population was studied elsewhere, and relevant results were also described elsewhere in more detail. In this systematic review, 31 articles that studied the effect of Se upon congenital anomalies were included [25,26,27,28,29,30,31,32,33,34,35,36,37,38,39,40,41,42,43,44,45,46,47,48,49,50,51,52,53,54,55]. Among those, 29 were retrieved by the aforementioned search algorithm in PubMed, Embase, and Cochrane Library, while 2 studies were retrieved through manual reference screening. The PRISMA flow-diagram depicts the article identification, screening, and inclusion process (Figure 1).

Of the 31 articles, 8 studied the occurrence of any congenital anomaly, including 20,815 pregnancies as participants in total. Nine articles studied neural tube defects, involving 12,188 pregnancies; however, three of these studies have recruited participants from the same population-based birth defects surveillance program in northern China, possibly including some same subjects. Orofacial clefts were studied in four articles with 1155 mother–child pairs, although there are concerns that the same subjects were included in these publications. Three other articles studied congenital heart defects involving 1807 mother–child pairs. Two studies examined chromosomal anomalies, in which 1242 participants were involved. Of the remaining five studies, one studied fetal alcohol syndrome including 41 pregnancies, one studied abdominal congenital anomalies based on data from 89,273 participants, two examined congenital limb anomalies with 551,547 participants, and lastly, congenital urinary tract anomalies were studied in one article which included 2150 mother–child pairs.

### 3.1. All/Any Congenital Anomaly

Eight studies examined concomitantly all congenital anomalies, six cohort studies, one case-control, and one RCT (Table 2, Tables S3 and S4) [25,26,27,28,29,30,31,32]. A wide range of geographical regions (Iran, Italy, USA, China, Israel, Saudi Arabia, South Korea), mostly urban areas, was covered. The sample size varied from 111 to 14,834, with both sample sizes noted in cohort studies, indicating the great difference in power between the included studies.

Two studies examined the effect of Se upon the occurrence of congenital anomalies through environmental exposure, measuring the levels of Se in the drinking water in the region where the participants resided throughout pregnancy [26,27]. Despite more than a ten-fold variation in drinking water Se concentrations between these studies and the application of confounder adjustment in only one study, both studies concluded that Se levels in drinking water were not significantly associated with the overall occurrence of congenital anomalies [26,27].

Five studies measured Se levels in body fluids, three in maternal venous blood, and two in maternal urine, while the timing of Se level assessment did vary significantly, from early pregnancy to prior to delivery. Two studies suggested that higher maternal venous blood Se concentrations may be associated with lower risk of congenital anomalies [30,32], whereas one study with lower statistical power reported no significant association [31]. In contrast, one study assessing maternal urinary Se excretion reported an association between higher urinary Se levels and an increased risk of congenital anomalies in a dose-dependent manner [28]. Notably, these findings were not consistent across different statistical models, and substantial concerns regarding residual confounding remain, as none of the studies adjusted their analyses for gestational age at the time of sampling. One double-blind RCT studied the effect of supplementation of pregnant women from the first trimester until delivery with 100 μg Se as Se yeast [25]. Although the Se levels in maternal venous blood (but not in cord blood) were significantly higher in the supplemented group compared to the non-supplemented, Se supplementation was not significantly associated with the risk of congenital anomalies. There are, however, significant concerns regarding risk of bias due to deviations from intended interventions in this RCT.

In conclusion, two studies involving 1609 participants reported a protective role of Se against congenital anomalies, five studies including 17,531 pregnancies in total found no significant effect, while one study of 1675 pregnancies reported a harmful effect. Overall, it is unlikely that environmental Se exposure or Se levels in maternal biological fluids affect the risk for offspring congenital anomaly. However, specific types of congenital anomalies need to be examined, as the co-existence of both protective and detrimental effects of Se on different congenital anomalies could lead to no evidence of effect when all congenital anomalies are examined concomitantly. With regard to certainty in the body of evidence, it is assessed as very low. Even though the majority of the included studies provided no indication of a significant association between maternal Se status during pregnancy and the risk of any fetal congenital anomaly, both protective and detrimental effects have been reported. Coherence was observed across studies evaluating environmental Se exposure and the RCT of Se supplementation, but not the studies measuring Se concentrations in maternal blood and urine. Concerns about risk of bias due to confounding are present for studies that did not implement statistical methods to reduce bias, and residual confounding—particularly by gestational age at sampling and smoking—may have influenced the findings of studies that adjusted for other covariates. Lastly, exposure indirectness was noted in five studies, either due to evaluating environmental Se exposure or obtaining biological samples after the development of congenital anomalies. We did not downgrade confidence in the body of evidence due to imprecision, despite the small sample size in some studies, which raises the possibility of type II error.

### 3.2. Neural Tube Defects

Nine case-control studies examined the relationship between Se and neural tube defects and involved in total a maximum of 12,118 pregnancies, of which 1311 were complicated by a neural tube defect (Table 3, Tables S3 and S4) [35,36,37,38,39,40,41,42,43]. The included studies were conducted in mainly rural regions of China (four studies), urban regions of Turkey (three studies), the UK (one study), and Canada (one study). The studies’ sample sizes ranged from only 28 [41] to 6420 pregnancies [36]. Two studies examined the effect of environmental Se exposure, while seven studies measured Se levels in biological samples (three in placental tissue, four in maternal blood, one in cord blood, and one in infant blood).

The effect of environmental Se exposure was explored by two studies measuring Se in drinking water [35] or Se levels in the soil of the area of residence throughout pregnancy [36]. Higher Se levels in drinking water were associated with higher risk of anencephaly in the unadjusted analysis, but this relationship was not significant after adjusting for potential confounders [35]. Similarly, no significant association was observed for Se levels in soil [36].

With regard to maternal Se levels that were examined in four studies, three studies reported a protective effect of Se against neural tube defects, while one study found no effect [38,40,41,42]. However, in all four studies, maternal blood samples were drawn after the first trimester, when neural tube defects develop [38,40,41,42]. In addition, one study observed a protective effect of Se only when leukocyte levels were considered, but no effect for plasma or erythrocyte levels [38]. Concerns for significant residual confounding are noted in the single study that found no association between maternal Se levels and neural tube defects, since neural tube defect cases had lower gestational age at sampling and higher maternal age, and these confounders were not taken into account in the statistical analysis [40].

Considering the three studies that used placental tissue to measure Se levels, samples were collected at the time of delivery or pregnancy termination, and sample size varied from 130 to 1001 [37,39,43]. Two studies reported that higher Se levels are associated with higher risk of neural tube defects [37,43], while one study of 130 participants found no significant association [39]. Notably, in this study, median Se concentrations in both affected and unaffected pregnancies were approximately four- to five-fold lower than those reported in the other two studies, which may indicate a detrimental effect of Se only when a cut-off placental concentration is exceeded [37,39,43].

Lastly, two studies have examined Se levels in the fetal compartment [40,42]. Lower infant plasma Se levels, but not cord blood Se levels, were associated with higher risk of neural tube defects [40,42]. However, confounding by maternal age and gestational age at sampling may have affected the results regarding cord blood Se levels [40].

In conclusion, there is no solid evidence to suggest that environmental Se levels can affect the risk of neural tube defects. Maternal Se levels may exert a protective effect, which is also supported by a study that examined infant blood Se levels. On the contrary, evidence from studies examining placental Se levels is limited, and suggests either no association or a potential detrimental effect at high placental Se concentrations, indicating that a U-shaped relationship cannot be excluded. The overall certainty in the body of evidence was rated as very low, primarily due to the aforementioned directional inconsistency and limited coherence across studies, the potential for residual confounding—particularly by gestational age in studies evaluating maternal Se levels—and exposure inconsistency. The latter reflects the assessment of environmental Se exposure or Se concentrations in biological samples collected after the first trimester of pregnancy, which represents the critical developmental window for neural tube defects.

### 3.3. Orofacial Clefts

The risk of orofacial clefts in relation to Se during pregnancy has been examined in four case-control studies from China, exploring the role of maternal blood levels, placental tissue levels, umbilical cord tissue levels, and umbilical blood levels (Table 4, Tables S3 and S4) [44,45,46,47]. After appropriate adjustment for confounding factors, placental tissue and umbilical cord tissue Se levels exerted a protective effect upon the risk of orofacial clefts in a dose-dependent manner [44,45]. In the analyses of maternal and umbilical cord blood Se levels, statistical significance was not reached, even though results were in the same direction with Se levels being lower in case of orofacial clefts [46,47]. To be noted, the study of umbilical cord blood Se levels had the lowest power and was the only one not controlling for any confounding factor in the study design or the statistical analysis [46].

The certainty in the body of evidence is regarded as moderate due to exposure indirectness. Three studies evaluated Se levels at the time of delivery [44,45,46], while one study used maternal blood samples obtained throughout pregnancy [47]; thus, the biologically relevant time period of the first trimester has not been directly reflected in any of the reviewed studies. Nonetheless, effect estimates were directionally consistent and reasonably precise, a dose-dependent association was observed regarding placental and umbilical samples, risk of bias was addressed, and there were no indications of publication bias.

### 3.4. Congenital Heart Defects

Congenital heart defects were examined in three case-control studies from China and the USA (Table 5, Tables S3 and S4) [48,49,50]. One study estimated environmental Se exposure via drinking water and reported a protective effect against the formation of congenital heart defects in a dose-dependent manner [48]. In line with these findings, lower maternal venous blood Se levels in the second and third trimester [49] and cord blood at birth [50] were associated with higher risk for congenital heart defects after adjusting for important confounders. On the contrary, higher Se levels in maternal hair samples were linked to an increased risk for congenital heart defects in adjusted analyses [50].

In conclusion, all three studies, involving a total of 1807–2112 pregnancies, concluded that Se has a protective effect against congenital heart defects, except for Se levels in hair samples, where the opposite relationship was observed. The certainty in the evidence presented above is moderate, due to downgrading for exposure indirectness. One study examined environmental Se exposure, while the other two studies measured Se levels in biological samples after the first trimester of pregnancy. Overall, the results demonstrated directional consistency and coherence, with the exception of findings derived from hair samples. Effect estimates were precise and a dose-response relationship between Se status during pregnancy and the risk of congenital heart defects was observed. Potential sources of bias were addressed in the statistical analyses, although results were not adjusted for smoking in the environmental exposure study, introducing the possibility of residual confounding [48].

### 3.5. Fetal Alcohol Syndrome (FAS)

One case-control study conducted in Finland has focused on FAS, by examining maternal venous blood and cord blood Se levels from 41 pregnant women (Tables S3 and S4) [51]. Compared to abstinent controls, maternal Se levels were higher in the case of FAS, but lower in unaffected by FAS drinking women; however, these differences were observed only during the second and third trimester. On the contrary, umbilical Se levels were lower in drinking mothers compared to controls, regardless of FAS [51]. The certainty in evidence is regarded as very low due to the small sample size leading to imprecision, and the lack of adjustment for potential confounders leading to very serious risk of bias. In addition, directional inconsistency was observed in results from umbilical and maternal biological samples.

### 3.6. Abdominal Congenital Anomalies

The risk of abdominal congenital anomalies has been examined among 89,273 pregnancies participating in a prospective cohort, the Japan Environment and Children’s Study (Tables S3 and S4) [52]. After adjusting for relevant confounders, no significant association with maternal venous blood Se levels was observed. However, an increased risk for omphalocele was reported for the highest quartiles of Se concentration, although there was no linear trend [52]. The Japan Environment and Children’s Study had a proper study design that enabled extensive statistical adjustment for potential confounders, and its large sample size produced stable effect estimates. However, no adjustment for gestational age at sampling was applied, leading to concerns regarding bias, and Se levels were measured during the second and third trimester rather than the first trimester, when the abdominal wall is developed; thus, indirectness of exposure is considered present. Consequently, certainty in evidence is low.

### 3.7. Congenital Limb Anomalies

Congenital limb anomalies have been examined in two highly powered studies, one prospective cohort embedded in the Japan Environment and Children’s Study (*n* = 90,163) [53] and one MR study (*n* = 461,384) (Tables S3 and S4) [54]. No statistically significant impact on the risk of congenital limb anomalies was observed in the observational study across all applied statistical methods, both crude and adjusted [53]. The results from the MR study were similar, which used single nucleotide polymorphisms as an instrumental variable (IV) [54].

The certainty of evidence is considered moderate due to risk of bias in the reviewed studies. In the observational study from Japan, no adjustment for gestational age was applied, while bias from additional direct effects between the IV and the outcome may be present at the MR study, owing to the IV being selected upon genome-wide association studies (GWAS) hits. Due to the inclusion of the MR study, which reflects well Se exposure during the first trimester of pregnancy when fetal limbs are developed, we did not downgrade the certainty of evidence due to exposure indirectness, even though the observational study used post-first trimester blood samples for Se level assessment.

### 3.8. Congenital Urinary Tract Anomalies

A case-control study embedded in the Japan Environment and Children’s Study examined congenital urinary tract anomalies, and measured maternal venous blood Se levels among 2150 participants during the second or third trimester (Tables S3 and S4) [55]. Applying extensive control for potential confounders, both by matching and statistical adjustment, a harmful effect of Se upon the risk of isolated congenital urinary tract anomalies was reported in a dose-response manner. However, this relationship was not observed when congenital urinary tract anomalies were combined with other congenital anomalies [55]. Given the appropriate study design of the Japan Environment and Children’s Study, the extensive adjustment for potential confounders, the acceptable precision of the effect estimates, and the statistical indications in favor of a dose-response relationship, the certainty of evidence is judged to be moderate, reflecting exposure indirectness, as Se levels assessment did not include the first trimester of pregnancy.

### 3.9. Chromosomal Anomalies

Lastly, congenital anomalies were also studied in the context of chromosomal anomalies by two research groups, both measuring Se levels in amniotic fluid during the second trimester of pregnancy (Tables S3 and S4) [33,34]. Both studies concluded that Se levels in amniotic fluid were not significantly different in the case of chromosomal anomalies, despite apparent methodological differences. The cross-sectional study from Poland has considerably lower statistical power (*n* = 156, of which 19 with chromosomal anomalies) and reported considerably higher Se levels (8 μg/L in pregnancies with chromosomal anomalies) [33]. In comparison, the nested case-control study from China involved 1086 participants (387 with chromosomal anomalies), and Se levels in pregnancies with chromosomal anomalies were 0.631 μg/L [34]. Although the included studies demonstrated broadly consistent findings, concerns regarding risk of bias due to confounding by gestational age and exposure indirectness due to measuring Se levels concomitantly with the outcome reduced our confidence. We, thus, rated the certainty of the evidence as low.

### 3.10. Quality Appraisal of the Included Studies

The two studies employing the most robust designs, namely, the RCT and the MR study, were both judged to be at high risk of bias (Table S4). For the RCT, high risk of bias was identified due to deviations from the intended interventions, with additional concerns related to the randomization process and the selective reporting of results [25]. In the MR study, the risk of bias was high for the domain of additional direct effects between IV and outcome, moderate for domains of weak instrument bias, genetic confounding bias, and participant selection, and low for other confounding bias; most of these concerns arose from incomplete reporting, as the MR study is currently available only as a preprint [54].

Among the cohort studies included in this review, the main concerns were related to comparability, as no study received the maximum of two stars, raising the possibility of residual confounding (Table S4). Selection issues were identified in studies in which the outcome may have been present at baseline, particularly when enrollment occurred after the first trimester. Outcome-related concerns arose from incomplete reporting of loss-to-follow-up rates.

Among the case-control studies included in this review, comparability-related concerns were noted, similar to those observed in the cohort studies (Table S4). Selection issues were identified due to the registry-based definition of cases, insufficient information to assess the representativeness of cases, and the use of hospital-based controls. Exposure-related concerns were primarily related to incomplete reporting of non-response rates in both cases and controls.

## 4. Discussion

In this systematic review of the literature, we explored the potential effect of Se exposure during pregnancy on the risk of congenital anomalies. When all congenital anomalies are concomitantly examined, it is unlikely that Se exerts any effect. Similarly, Se is unlikely to affect the risk for abdominal or limb anomalies, chromosomal anomalies, or FAS. On the contrary, observational data support that higher maternal Se levels may be harmful for the development of urinary tract system, but protective against neural tube defects and heart defects. Notably, a detrimental effect of Se in high placental concentrations may be present for neural tube defects, raising the possibility of a U-shaped association. Lastly, Se may exert a protective effect against orofacial clefts, but supporting evidence is limited.

### 4.1. Selenium (Se) and Teratogenesis: Potentially Implicated Biological Mechanisms

Congenital anomalies are defined as structural or functional defects that occur during the prenatal period and can be identified at birth or later in life. Every year, around 295,000 newborns die before reaching the fourth week of life due to congenital anomalies and relevant complications, while the surviving infants suffer an important decline in their quality of life (measured in disability adjusted life years, DALYs), as well as stigma and discrimination from their local communities [56]. Identified risk factors for congenital anomalies can be divided into modifiable and non-modifiable. Specifically, gene mutations, parental chromosomal anomalies, multi-factor genetic predisposition, as well as advanced maternal age and history of a previous pregnancy affected by birth defects are some well-known and non-modifiable risk factors [56]. Environmental exposures also play an influential role, including specific viruses, alcohol and tobacco use during pregnancy, environmental toxic agents, and nutrient deficiencies [56]. Among environmental exposures, Se is a trace element with well-established beneficial properties, but is also linked to harmful effects upon human health and development when exposure is below or above specific levels.

Organogenesis refers to the process of proliferation and differentiation of tissues into organs, which occurs during weeks three to eight post-fertilization. During the third week, three distinctive cell layers are formed through the process of gastrulation, namely, the ectoderm, the mesoderm, and the endoderm [57]. Each cell layer gives form to specific organ systems through further differentiation and growth. By week eight, the process of organogenesis is completed, and by week nine, organ systems start to form more mature anatomical structures during the fetal period [57]. Organogenesis is strictly regulated by molecular and biochemical processes, so the fetus is more susceptible to teratogenic environmental factors during that period, leading to defects and malformations [58]. Oxidative stress is a dynamic equilibrium between reactive oxygen species (ROS) and antioxidant factors (enzymatic or non-enzymatic) [58]. At low concentrations, ROS seem to have a positive impact on cellular functions, as they regulate several enzymes and transcription factors. At higher concentrations, oxidative stress disrupts vital signaling pathways, leading to macromolecular damage which consequently harms the embryo’s homeostasis, leading to congenital malformations or even fetal death and abortion [58]. Specifically, ROS and free radicals constitute partially reduced intermediates that occur through aerobic metabolism and can cause damage through various molecular pathways. Reactive oxygen species can have a harmful effect upon cell membrane integrity and flexibility, as the lipid-rich membrane can become a target of lipid peroxidation [59]. Protein and enzymatic stability and integrity can also be harmed by oxidative stress mediators, as ROS can cause oxidation of specific amino acid side chains, in a process called protein carbonylation [43]. Lastly, ROS affect DNA strands in multiple ways, such as base modifications or strand breaks, causing mutagenesis or cell apoptosis [59]. Pregnancy is normally a period of increased oxidative stress, especially during the first trimester, when the production of hyperoxide anions is enhanced. Throughout pregnancy, possibly as a response to the oxidative state, the expression of antioxidant enzymes is also increased. Expression of superoxide dismutase, catalase, and GPx is 150% higher during the last 15% of gestation [58,60].

More specifically, oxidative stress has been linked to congenital heart defects, the most common congenital malformation, affecting nearly one percent of newborns worldwide and contributing strongly to newborn morbidity and mortality [61]. Women who carry offspring with congenital heart defects have lower concentrations of anti-oxidative markers (e.g., reduced glutathione), higher concentrations of oxidative markers (e.g., homocysteine), and lower oxidative balance score (a dietary index of oxidative balance) compared to non-affected pregnancies [61,62]. Oxidative damage in the premise of elevated production of ROS has also been proposed as a possible link between pre-gestational diabetes and the high occurrence of congenital heart defects in the infants of these patients, as studies have shown a lower occurrence of these malformations after supplementation with antioxidant substances [63]. This observation is strengthened by animal data showing that female rats with drug-induced pre-gestational diabetes have reduced risk of giving birth to a pup with congenital heart defect when they exercise, which lowers ROS and thus oxidative stress [64].

Similar observations are made about oxidative stress during gestation and neural tube defects, another quite common type of congenital anomaly. Oxidative stress parameters (thiol-disulfide homeostasis parameters and ischemia-modified albumin) have been measured at higher concentrations in the serum of pregnancies affected by neural tube defects [65]. These findings are also supported by animal data, showing that treatment of mice with benzo[a]pyrene (BaP) increases the risk of neural tube defects in offspring and the expression of genes related to oxidative stress, while suppressing GPx [66]. Furthermore, neural tube defects can be induced in chick embryos by homocysteine treatment, which increases the intracellular oxidative species in offspring, while decreasing in the activity of antioxidant enzymes (e.g., GPx) [67].

The potential influence of Se on the process of organogenesis could be explained under the scope of selenium’s antioxidant properties. Selenium plays a pivotal role in the regulation of many antioxidant enzymes, such as GPx, one of the most abundant in the human body, owning an important place within antioxidant factors. Thus, Se deficiency leading to reduced levels of Se-dependent antioxidant enzymes can contribute to the observed increased risk of congenital anomalies, such as neural tube defects, heart anomalies, and orofacial clefts.

The potential harmful effect of Se upon organ formation could also be examined under the scope of thyroid hormone regulation during pregnancy. Selenium protects the thyroid gland from oxidative damage through GPx, while controlling the conversion of T4 into the active T3 and the formation of the inactive compound through the incorporation into DI. Consequently, Se depletion or excess could be linked to several thyroid pathologies [68]. Thyroid hormone balance during fetal development is crucial, as thyroid hormones (T3 and T4) regulate infant growth and especially nervous system development. Imbalance in the levels of these hormones may lead to impaired cognitive, motor, and sensory functions in the infant [69], as they seem to control neuron proliferation, differentiation, and migration, ensuring the formation of organized neural circuits in the developing brain [70]. Regarding the formation of congenital anomalies, existing evidence is rather conflicting. In a prospective cohort study from China, clinical and subclinical hypothyroidism was associated with congenital malformations, specifically congenital heart defects, poor vision development, and musculoskeletal malformations, while clinical hyperthyroidism was associated with hearing dysplasia [71]. Supporting evidence from animal model studies have shown that the depletion of DI3 in mice can lead to brain and cranial malformations, hydrocephalus, choanal atresia, and cleft palate through secondary hyperthyroidism [69]. On the contrary, a non-significant relationship between thyroid hormone levels in mothers and the occurrence of congenital malformations in their offspring has been observed both in case-control [72] and cohort studies [73].

Another important interaction is that between Se and folate, as folate deficiency is specifically associated with the development of neural tube defects. Folate refers to the different forms of vitamin B9, and is an important part of the one-carbon metabolism. Folate metabolites serve as carbon donors for the conversion of homocysteine into methionine, an amino acid used for protein synthesis and as a co-enzyme in DNA methylation processes [74]. Through epigenetic regulation, folate alters genetic pathways crucial for infant development [75,76], while reducing the levels of homocysteine in maternal circulation, a toxic amino acid linked to neural system deterioration [75]. Folate dietary supplementation with 400 μg/day periconceptionally and during pregnancy has been an established guideline for decades, significantly reducing neural tube defect occurrence [74]. The relationship between Se and folate metabolism has been explored to explain the link between Se and neural tube defects through the biological effects of folate. A synergistic action of Se and folate has been indicated, as GPx activity is enhanced in pups born from folate-deficient dams supplemented with Se [77]. In addition, the normal increase in homocysteine levels caused by folate deficiency is ameliorated by higher Se levels in folate-deficient rats supplemented with Se [78].

Selenium may also exert protective against congenital anomalies properties through interacting with other elements and thus ameliorating a possible harmful effect. These interactions have gained more importance as massive industrialization has led to extended environmental chemical contamination. An important and dangerous contaminant is, for example, mercury (Hg), a non-essential element, which, mainly through air pollution, deposits in water environments, where it takes the form of methyl-mercury and reaches humans mainly via fish consumption [79,80]. In high concentrations, Hg is a well-established neurotoxicant, causing disturbances in memory and attention, as well as defects in motor, language, and visual skills [81]. Mercury can cross the placenta and the blood–brain barrier and tends to accumulate in brain tissue, depicting a possible threat for infant neurodevelopment, especially as concentrations in cord blood generally seem to be higher than in maternal blood [82]. Significantly higher levels of Hg in maternal blood of neural tube cases than in normal controls have been observed [42], while a significant association with congenital heart defects was reported in a systematic review and meta-analysis [83]. Methyl-mercury shows high affinity for Se; the formation of Hg–Se compounds or the integration of Hg in selenoproteins has been proposed as a protective mechanism of Se against Hg accumulation and thus toxicity [79]. Similar interactions have been studied for Se and arsenic, Se and cadmium [84], as well as copper and iron [85].

### 4.2. Supportive Data Emerging from Animal Model Studies

Several animal model studies have elucidated the beneficial role of Se in healthy embryonic development. Selenium supplementation in pregnant goats during gestation improves total antioxidant capacity and levels of reproductive and thyroid hormones [86]. Selenium deficiency has been linked to reduced antioxidant enzyme activity and increased protein oxidation [87], leading to increased mortality at birth and impaired growth parameters [88]. On the contrary, environmental exposure to high Se concentrations in the ecosystem has been related to deformities in several animal species, while animal model studies have linked high Se levels to congenital malformations. Severe deformities, such as skeletal and eye malformations, have been observed in fish living in a contaminated ecosystem in North Carolina with 130-fold higher Se concentration compared to non-contaminated reference ecosystems [89]. Similarly, eye, limb, and other congenital malformations have been reported in aquatic bird embryos living in Se-contaminated irrigation drainage evaporation ponds [90]. In line with these observational data, animal model studies of Se supplementation corroborate the aforementioned observations. Low survival rates of the embryos, reduced growth, and limb and heart malformations were observed in chick embryos after injecting various concentrations of selenous acid in the eggs [91]. Similarly, rat embryos cultured with Se compounds in various concentrations demonstrate high occurrence of head malformations (hypoplastic telencephalon, hypoplastic olfactory system), with inorganic compounds being more teratogenic than organic [92]. Craniofacial and heart congenital malformations have also been reported in medaka fish embryos birthed by mothers supplemented with Se nanoparticles [93]. These findings collectively indicate that Se is linked to specific deformities that influence a range of animal species, including birds, fish, and mammals, at least in a high exposure setting, while Se deficiency has been also shown to negatively affect gestation in animal models. Therefore, a U-shaped association may also be the case for humans, underlining the importance of maintaining Se levels within the relatively narrow recommended range.

### 4.3. Public Health and Clinical Practice Implications

Despite the growing evidence about the importance of Se levels for the maintenance of a healthy pregnancy and thus the development of a healthy embryo, the existing guidelines regarding Se intake, optimal Se levels, and Se supplementation in pregnancy are scarce. According to the World Health Organization, the recommended daily intake of Se for pregnant women is 60 μg for all age groups and 70 μg for lactating women, while the tolerable upper intake levels are set at 400 μg [94,95]. According to the National Health and Nutrition Examination Survey (NHANES) study (2017–2018), the daily Se intake of adult women aged 20–39 was 97 μg/day [96]. Regarding Se levels in biological samples, these have been consistently shown to differ between different populations, between non-pregnant and pregnant individuals, as well as by pregnancy trimester. Selenium levels can be assessed in blood (whole blood, plasma, or erythrocytes), urine, hair, or nails [97]. Even when measured in the same biological sample, significant variation is present. For example, a mean Se concentration of 113.1 μg/L in the serum of women in Finland was reported, compared to 67.1 μg/L in the UK [97]. Generally, Se levels in pregnant women are significantly lower compared to non-pregnant women, while a decline is observed at the second trimester, third trimester, and during delivery [97,98]. The increased demand and usage of antioxidant factors by the developing embryo contributes to the observed decrease in Se levels in maternal serum throughout pregnancy. Selenium supplementation and monitoring during pregnancy is not a part of obstetrical daily clinical practice, as it is for folic acid or iron, although 40% of prenatal supplements do contain Se at a mean concentration of 70 μg [96]. A possible explanation for this lack of guidance is the need for a more systematic approach regarding the effects of Se on pregnancy that would demonstrate the health impact observed both in case of Se deficiency and Se over-consumption. Considering the occurrence of congenital anomalies and their impact upon children’s mortality and long-term quality of life, the exact effect of different Se levels upon the formation of specific congenital anomalies should be elucidated, for specific guidelines to be formulated.

### 4.4. Strengths and Limitations of This Study

In this systematic review, heterogeneity in the results of the reviewed studies has been noted and may be explained by differences in sample size and statistical power, differences in statistical methodologies applied and potential confounding factors that were accounted for, differences in timing of sampling during pregnancy, biological samples examined, and different concentration ranges observed in study populations. The developing fetus is more sensitive to environmental exposures, including changes in Se concentration, during the first trimester when organogenesis occurs; thus, sampling during that period is of actual clinical relevance. However, most of the studies measured Se during late pregnancy or even at delivery. It remains, however, unclear how well late pregnancy Se levels reflect the Se levels during periconception and the first trimester, while sampling after the development of congenital anomalies raises concerns regarding reverse causality. Furthermore, different organ systems may have different toxicity thresholds given that Se is a trace element with a narrow toxicity window, which is of importance during organogenesis when the susceptibility of the growing fetus to environmental exposure rises. With regard to different biological samples examined, Se concentration is expected to differ between different samples (for example maternal venous blood and maternal urine), but also between maternal, placental, cord, and infant samples. Selenium concentration in cord blood is observed by some studies to be lower than maternal venous blood, which is expected, as Se transportation through the placenta is based on a concentration gradient [99]. Other studies, however, reported a higher concentration in cord blood [100]. Other biological samples, such as hair and nails, reflect the Se levels long-term and not exclusively during the periconception and pregnancy period, making interpretation of results harder. Lastly, the concentration range observed in each study will naturally influence the results, as both Se deficiency and Se excess may negatively affect the risk for congenital anomalies, while a protective effect of Se may be present only within the optimal Se range. For instance, in studies reporting an increased risk of neural tube defects with elevated Se levels in placental tissue [37,43], significantly higher placental Se levels were observed than in studies reporting a non-significant association [39].

This systematic review aims to provide a comprehensive overview of current research data on the role of Se exposure during pregnancy in development of fetal congenital anomalies. An extensive search strategy and strict criteria for inclusion of clinical studies have been applied to ensure the validity and accuracy of our conclusions, while PRISMA guidelines for systematic review reporting have been followed to ensure transparency in reporting. However, this systematic review is also subject to several limitations. The protocol for this review has not been registered; however, the SEduP protocol will be made available upon request. The number of clinical studies addressing the occurrence of congenital anomalies in relation to Se exposure is rather small, and strength of evidence is limited by study design. Only one reviewed study was a double-blind RCT that recruited 125 pregnant women, thus being severely underpowered in the context of congenital anomalies, and the single MR study identified is currently accessible only as a preprint. The majority of data (21/31 studies) arose from case-control studies or cross-sectional studies, while eight cohort studies have also been reviewed. The period of data collection also varied widely, but the age of data should not influence the accuracy of the results. Nonetheless, it may have affected the observed average Se concentration in each study, since regulations for water Se concentrations have been applied during the last 30 years and changes in activities, such as mining, agriculture, and industrial activity that affect Se concentration in the environment have occurred [101]. In addition, European studies are under-represented, with only 5 out of 31 studies conducted in European populations. Lastly, a formal meta-analysis of the results of reviewed studies was not feasible, owing to the heterogeneity in biological sample studies, timing of sampling, and statistical methodologies applied.

### 4.5. Identified Research Gaps and Future Directions

Although our systematic review could not provide a definite answer on the role of Se in the development of congenital malformations, specific research gaps have been identified and directions for future clinical studies are provided, so that a specific conclusion with clinical relevance may be reached. With regard to Se levels, future studies should elucidate the differences between maternal, placental, and fetal compartment and whether these differ between affected and non-affected pregnancies. Notably, sampling should be performed at similar gestational age both for pregnancies with identified congenital anomalies and controls. Thus, when induced abortions due to congenital anomalies are studied, the appropriate control group should not consist of term pregnancies without any pathology, but of elective abortions of similar gestational age. Moreover, studies assessing maternal selenium status in relation to congenital anomaly risk should prioritize exposure assessment during the first trimester of pregnancy, which represents the critical window for organogenesis, to limit the potential of reverse causality. This remains methodologically challenging, as many women initiate prenatal care late in the first trimester or beyond. Given that the prevalence of congenital anomalies is higher among women of advanced maternal age, who are also more likely to conceive using assisted reproductive technologies, future research should explore whether pregnancies achieved through in vitro fertilization and embryo transfer are differentially affected by selenium exposure. This may have important implications for risk stratification and targeted preventive strategies. Of utmost importance is the conduction of RCTs with different Se dosages and concomitant monitoring of Se dietary intake. Such studies can provide high-quality interventional data and change clinical practice, but should be powered enough to detect differences in congenital anomalies taking into consideration the relatively low incidence of each type of them. In addition, MR represents a valuable complementary approach for assessing causality in observational associations. Nevertheless, careful selection of IVs is essential, avoiding reliance solely on GWAS hits to minimize bias due to pleiotropy, and ensuring that associations between IVs and potential confounders are excluded to reduce genetic confounding. Lastly, it is important for future studies to account for the impact of different levels of environmental Se exposure and maternal baseline Se levels, so that meaningful conclusions can be extrapolated and applied to different populations.

## 5. Conclusions

In this systematic review, higher maternal Se exposure during pregnancy was associated with an increased incidence of urinary tract anomalies and a decreased incidence of neural tube defects, congenital heart defects, and orofacial clefts, while no significant associations were observed with overall congenital anomalies, abdominal wall and limb defects, chromosomal abnormalities, or FAS. Moreover, interpretation of reviewed studies suggested a potential U-shaped relationship between Se status and the risk of congenital anomalies. Although the available evidence indicates that Se may play a biologically relevant role in organogenesis, the confidence in the body of evidence ranges from very low to moderate, while the reported associations are derived primarily from observational studies and are subject to residual confounding and the potential for reverse causality, warranting cautious interpretation. Given that congenital anomalies represent a major contributor to neonatal morbidity, mortality, and long-term impairment of quality of life, further well-designed prospective studies are required to establish a scientific consensus, define optimal maternal Se levels, and provide evidence-based, high-quality recommendations for Se supplementation during pregnancy.

## Figures and Tables

**Table 1:** The search strategy in PubMed.

(“pregnancy”[MeSH Terms] OR “pregnan *”[Title/Abstract] OR “gestation”[Title/Abstract] OR “prenatal”[Title/Abstract] OR “intrauterine”[Title/Abstract] OR “in utero”[Title/Abstract] OR “perinatal”[Title/Abstract] OR “postnatal”[Title/Abstract])
AND
(“selenium”[MeSH Terms] OR “selenium”[Title/Abstract])
NOT
(“animals”[MeSH Terms] NOT “humans”[MeSH Terms])

* indicates truncation used to capture multiple word variants sharing the same root.

**Table 2:** Overview of included studies examining Se in relation to any congenital anomaly.

Study	Se Exposure	Main Findings	Quality Appraisal (NOS Scale or RoB 2 Tool)		
Environmental Se exposure					
Vinceti et al. 2000 [26]	Se levels in drinking water Exposed: 7–9 μg/L Unexposed: 1 μg/L	Se levels were not significantly associated with the risk of congenital anomalies.	Selection: ✸✸✸✸	Comparability: -	Outcome: ✸✸✸
Aschengrau et al. 1993 [27]	Median Se levels in drinking water: 0.1 μg/L	Se levels were not significantly associated with the risk of congenital anomalies, although some associations were observed for specific systems (central nervous system, integument).	Selection: ✸✸✸	Comparability: ✸✸	Exposure: ✸✸
Se supplementation					
Boskabadi et al. 2010 [25]	Supplementation of 100 μg Se daily as selenium yeast vs. placebo yeast tablets	Se supplementation was not significantly associated with the risk of congenital anomalies.	High risk of bias due to deviations from intended interventions		
Maternal Se levels					
Hammouda et al. 2013 [30]	Mean (SD) Se levels in venous blood Cases: 93.0 (32.0) μg/L Controls: 111.2 (30.2) μg/L	Higher Se levels were associated with lower risk of congenital anomalies. Se levels were however lower in cases of central nervous system and genitourinary system anomalies.	Selection: ✸✸✸✸	Comparability: -	Outcome: ✸✸
Choi et al. 2016 [31]	Median (IQR) Se levels in venous blood: 94.0 (89.0–101.0) μg/L	Se levels were not significantly associated with the risk of congenital anomalies.	Selection: ✸✸✸	Comparability: -	Outcome: ✸✸✸
Zhang et al. 2025 [32]	Mean (SD), [IQR] Se levels in venous blood: 110.321 (42.221), [83.333–138.184] μg/L	Higher Se levels were associated with lower risk of congenital anomalies. However, the statistical significance depended on the statistical method applied.	Selection: ✸✸✸	Comparability: ✸	Outcome: ✸✸
Lv et al. 2024 [28]	Median (IQR) Se levels in urine: 10.97 (8.38–14.39) μg/L	Higher Se levels were associated with higher risk of congenital anomalies. In some analyses, paternal Se should also be high for this effect to be observed.	Selection: ✸✸✸✸	Comparability: ✸	Outcome: ✸✸
Karakis et al. 2021 [29]	Median (95% CI) Se levels in urine: 19.58 (13.73–27.91) μg/L	Se levels were not significantly associated with the risk of congenital anomalies.	Selection: ✸✸✸	Comparability: ✸	Outcome: ✸✸✸

✸ indicate the number of criteria met within each Newcastle-Ottawa Scale (NOS) domain.

**Table 3:** Overview of included studies examining Se in relation to neural tube defects.

Study	Se Exposure	Main Findings	Quality Appraisal (NOS Scale)		
Environmental Se exposure					
Elwood et al. 1981 [35]	Mean Se levels in drinking water Cases (anencephalus): 3.20 μg/L Controls: 3.09 μg/L	Higher Se levels were associated with higher risk of anencephalus, but this relationship was not preserved after adjusting for possible confounders.	Selection: ✸✸✸	Comparability: ✸	Exposure: ✸✸
Huang et al. 2011 [36]	Mean Se levels in soil: 0.16 mg/g	Se levels were not significantly associated with the risk of neural tube defects.	Selection: ✸✸✸✸	Comparability: -	Exposure: ✸✸
Maternal Se levels					
Hinks et al. 1989 [38]	Mean (95% CI) Se levels in plasma Cases: 70.3 (63.2–77.4) μg/L Controls: 78.2 (74.2–82.1) μg/L	Se levels were not significantly associated with the risk of neural tube defects.	Selection: ✸✸	Comparability: -	Exposure: ✸✸
Zeyrek et al. 2009 [40]	Mean (SD) Se levels in plasma Cases: 46.8 (26.4) μg/L Controls: 47.6 (20.6) μg/L	Se levels were not significantly associated with the risk of neural tube defects.	Selection: ✸✸	Comparability: ✸	Exposure: ✸✸
Cengiz et al. 2004 [41]	Mean (SD) Se levels in plasma Cases: 55.16 (11.3) μg/L Controls: 77.4 (5.5) μg/L	Lower Se levels were associated with higher risk of neural tube defects.	Selection: ✸✸	Comparability: ✸	Exposure: ✸✸
Demir et al. 2019 [42]	Mean (SD) Se levels in plasma Cases: 55.4 (7) μg/L Contros: 79.9 (8) μg/L	Lower Se levels were associated with higher risk of neural tube defects.	Selection: ✸✸✸	Comparability: -	Exposure: ✸✸
Hinks et al. 1989 [38]	Mean (95% CI) Se levels in erythrocytes Cases: 4.39 (3.67–5.11) nmol/g Hb Controls: 4.85 (4.63–5.07) nmol/g Hb	Se levels were not significantly associated with the risk of neural tube defects.	Selection: ✸✸	Comparability: -	Exposure: ✸✸
Hinks et al. 1989 [38]	Mean (95% CI) Se levels in leukocytes Cases: 0.92 (0.81–1.04) pmol/10^6^ cells Controls: 1.28 (1.18–1.39) pmol/10^6^	Lower Se levels were associated with higher risk of neural tube defects.	Selection: ✸✸	Comparability: -	Exposure: ✸✸
Placental Se levels					
Jia et al. 2025 [43]	Median (IQR) Se levels Cases: 1300 (1180–1470) ng/g Controls: 1060 (950–1160) ng/g	Higher Se levels were associated with higher risk of neural tube defects.	Selection: ✸✸	Comparability: ✸	Exposure: ✸✸
Yin et al. 2020 [37]	Median (IQR) Se levels Cases: 1308.7 (1162.8–1466.7) ng/g Controls: 1142.5 (1013.1–1285.9) ng/g	Higher Se levels were associated with higher risk of neural tube defects, in a dose-dependent manner.	Selection: ✸✸	Comparability: ✸✸	Exposure: ✸✸
Liu et al. 2013 [39]	Median (IQR) Se levels Cases: 239.38 (207.92–284.86) ng/g Controls: 229.54 (202.78–261.22) ng/g	Se levels were not significantly associated with the risk of neural tube defects.	Selection: ✸✸	Comparability: ✸✸	Exposure: ✸✸
Cord Blood Se levels					
Zeyrek et al. 2009 [40]	Mean (SD) Se levels Cases: 42.2 (21.9) μg/L Controls: 39.9 (20) μg/L	Se levels were not significantly associated with the risk of neural tube defects.	Selection: ✸✸	Comparability: ✸	Exposure: ✸✸
Infant Se levels					
Demir et al. 2019 [42]	Mean (SD) Se levels Cases: 52.9 (0.6) μg/L Controls: 70.3 (0.6) μg/L	Lower Se levels were associated with higher risk of neural tube defects.	Selection: ✸✸✸	Comparability: -	Exposure: ✸✸

✸ indicate the number of criteria met within each Newcastle-Ottawa Scale (NOS) domain.

**Table 4:** Overview of included studies examining Se in relation to orofacial clefts.

Study	Se Exposure	Main Findings	Quality Appraisal (NOS Scale)		
Maternal venous blood Se levels					
Yin et al. 2020 [47]	Median (IQR) Se levels Cases: 89.201 (73.216–113.442) μg/L Controls: 93.304 (75.610–111.574) μg/L	Se levels were not significantly associated with the risk of orofacial clefts.	Selection: ✸✸	Comparability: ✸✸	Exposure: ✸✸
Placental Se levels					
Pi et al. 2019 [44]	Median (IQR) Se levels Cases: 1030 (900–1130) μg/g Controls: 1110 (1020–1240) μg/g	Higher Se levels were associated with lower risk of orofacial clefts in a dose-dependent manner.	Selection: ✸✸✸✸	Comparability: ✸✸	Exposure: ✸✸
Umbilical Cord Se levels					
Wei et al. 2024 [46]	Median (IQR) Se level in cord blood Cases: 91.288 (75.295–111.622) μg/L Controls: 94.159 (74.193–113.254) μg/L	Se levels were not significantly associated with the risk of orofacial clefts.	Selection: ✸✸✸	Comparability: -	Exposure: ✸✸
Ni et al. 2019 [45]	Median (IQR) Se levels in cord tissue Cases: 0.52 (0.46–0.60) μg/g Controls: 0.56 (0.50–0.62) μg/g	Higher Se levels were associated with lower risk of orofacial clefts, in a dose-dependent manner.	Selection: ✸✸✸✸	Comparability: ✸✸	Exposure: ✸✸

✸ indicate the number of criteria met within each Newcastle-Ottawa Scale (NOS) domain.

**Table 5:** Overview of included studies examining Se in relation to congenital heart defects.

Study	Se Exposure	Main Findings	Quality Appraisal (NOS Scale)		
Environmental Se exposure					
Zierler et al. 1988 [48]	Median Se level in drinking water 0.00 mg/L Highest detected level: 0.01 mg/L	Higher Se levels were associated with lower risk of congenital heart defects in a dose-response manner (especially coarctation of the aorta).	Selection: ✸✸	Comparability: ✸	Exposure: ✸✸
Maternal Se levels					
Ou et al. 2017 [49]	Median (IQR) Se levels in plasma Cases: 172.90 (153.87–192.23) μg/L Controls: 186.47 (172.45–207.34) μg/L	Higher Se levels were associated with a lower risk of congenital heart defects (especially conotruncal defects, septal defects, and right ventricle outflow tract obstruction).	Selection: ✸✸✸	Comparability: ✸✸	Exposure: ✸✸✸
Guo et al. 2019 [50]	Median (IQR) Se levels in hair samples Cases: 0.58 (0.5–0.73) ng/mg Controls: 0.58 (0.4–0.71) ng/mg	Higher Se levels were associated with higher risk of congenital heart defects.	Selection: ✸✸✸	Comparability: ✸✸	Exposure: ✸✸
Cord Blood Se levels					
Guo et al. 2019 [50]	Median (IQR) Se levels Cases: 33.76 (16.43–36.36) μg/L Controls: 37.68 (27.49–47.3) μg/L	Higher Se levels were associated with lower risk of congenital heart defects.	Selection: ✸✸✸	Comparability: ✸✸	Exposure: ✸✸

✸ indicate the number of criteria met within each Newcastle-Ottawa Scale (NOS) domain.

## Data Availability

No new data were created or analyzed in this study. Data sharing is not applicable to this article.

## Supplementary Materials

The following supporting information can be downloaded at: https://www.mdpi.com/article/10.3390/nu18030479/s1, Table S1: The search strategy in Embase; Table S2: The search strategy in Cochrane Library; Table S3: Summary of findings table of the reviewed studies; Table S4: Quality appraisal of the included studies; Table S5: The PRISMA 2020 checklist for abstracts; Table S6: The PRISMA 2020 checklist.
